# Fine‐Scale Movement Data Reveal Primarily Surface Foraging and Nocturnal Flight Activity in the Endangered Bermuda Petrel

**DOI:** 10.1002/ece3.71647

**Published:** 2025-06-30

**Authors:** Paolo Becciu, Allison Patterson, Carina Gjerdrum, Jeremy Madeiros, Letizia Campioni

**Affiliations:** ^1^ Department of Ecology and Evolution University of Lausanne Lausanne Switzerland; ^2^ Ornis Italica Roma Italy; ^3^ Swiss Ornithological Institute Sempach Switzerland; ^4^ Wildlife Research Division Environment and Climate Change Canada Dartmouth Nova Scotia Canada; ^5^ Canadian Wildlife Service Dartmouth Nova Scotia Canada; ^6^ Department of Conservation Services Ministry of the Environment Flatts Bermuda; ^7^ MARE—Marine and Environmental Sciences Centre/ARNET—Aquatic Research Network Ispa—Instituto Universitário Lisboa Portugal; ^8^ Departamento de Biología de la Conservación y Cambio Global Estación Biológica de Doñana, Consejo Superior de Investigaciones Científicas (EBD‐CSIC) Sevilla Spain

**Keywords:** accelerometer, activity budget, diving behaviour, dynamic‐soaring, flight mode, gadfly petrel, mesopelagic prey, *Pterodroma*

## Abstract

Foraging behaviour plays a fundamental role in animal fitness and population dynamics., particularly for central‐place foragers like breeding seabirds. Among Procellariiform seabirds, petrels exhibit a wide range of foraging strategies finely tuned to the patchy and unpredictable distribution of resources. The extent and remote nature of their foraging grounds makes direct observation of foraging behaviour impractical, thereby requiring the use of remote tracking technologies. We deployed miniaturised multi‐sensor biologgers and collected fine scale movement data to investigate the at‐sea behaviours of the Bermuda petrel *Pterodroma cahow* , a poorly studied and highly threatened gadfly petrel, specialised on mesopelagic prey. GPS‐tracking data revealed extensive foraging trips (mean ± SD: 1207 ± 305 km), in consistent directions, over remote oceanic regions. Time‐depth‐recorders provided new insights into Bermuda petrel feeding techniques suggesting that the meso‐bathypelagic prey targeted by petrels must be available in the very upper layer of the water surface, given their very limited diving activity (maximum dive depth of 1.57 m). We identified three flight‐related and three water‐associated behaviours using supervised classification approach to classify behaviour from tri‐axial acceleromtetry. Flying behaviours reflected the expected dynamic soaring flight strategy of Procellariiformes; individuals spent more than 75% of their time in flight (dynamic soaring and flap‐gliding) with dynamic soaring flight being the most common behaviour under all conditions. The behaviour classified as ‘Intensive flight’ was infrequently observed but could indicate aerial dipping, a characteristic foraging technique of *Pterodroma* species. The remaining time was spent in three water behaviours: active, inactive and intensive, with the latter being less common but thought to reflect scavenging and prey seizing. Flight increased during dusk and in the night, highlighting greater flight activity during night compared to the day, while water behaviours were more common during the day. While some of our findings may require further validation to confirm their relevance to foraging behaviour, our work offers new and valuable insights to consider when assessing the ecological needs of this endangered species and its potential vulnerability to offshore anthropogenic activities.

## Graphical abstract

This study examined the at‐sea foraging behaviour of the Bermuda petrel ( *Pterodroma cahow* ), a highly threatened gadfly petrel, using GPS loggers, time‐depth recorders, and accelerometers. Findings revealed extensive foraging trips, surface feeding, and pronounced nocturnal flight activity, offering valuable insights into the behavioural ecology of poorly studied Pterodroma petrels.

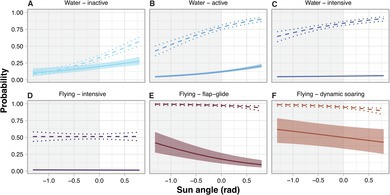

## 1 Introduction

Foraging is a fundamental component of animal behaviour with direct implications for individual fitness, survival, and population demography (Stephens et al. 2007). Maximising foraging efficiency is critical for predators that must trade off spatiotemporal constraints with their energetic needs. This challenge is typical in central place foraging scenarios such as the one faced by many seabirds during the breeding season (Orians and Pearson 1979). For instance, during the incubation period, seabirds must alternate between self‐feeding and taking over incubation duties from their partner. Consequently, the time available for one individual to replenish its energy reserves is constrained by the fasting endurance of its incubating partner, which in some species can extend for up to 3 weeks (Lovette and Fitzpatrick 2016).

Procellariiform seabirds, such as petrels and albatrosses, have evolved diverse foraging strategies (Schreiber and Burger 2001) to navigate vast and dynamic pelagic environments while meeting the constraints of central‐place foraging. These strategies are particularly effective in environments where prey is sparsely and unpredictably distributed, requiring individuals to balance time spent in transit, exploratory searching and exploiting available resources (Young and VanderWerf 2023). A key factor shaping these foraging strategies is the species' flight style, which is closely linked to energy expenditure and prey acquisition. For instance, albatrosses, shearwaters and many petrels have evolved morphological and aerodynamic adaptations that optimise their ability to locate and capture prey over vast distances, primarily relying on dynamic soaring, an energy‐efficient flight technique that enables them to exploit wind gradients over the ocean to travel with minimal energetic cost (Pennycuick 2002; Sachs et al. 2012). However, not all species rely on this strategy to the same extent. Some alternate between dynamic soaring and more energetically demanding flapping flight or rely predominantly on flapping, both of which, in turn, influence their movement patterns and diel activity budgets (Bonnet‐Lebrun et al. 2021).

Differing foraging strategies give rise to distinct diel activity patterns. Large seabirds, such as albatrosses, are predominantly diurnal foragers, with intense flying and feeding activities concentrated during daylight hours (Fernández and Anderson 2000; Phalan et al. 2007; Pajot et al. 2021). In contrast, several small petrels, including the Bulwer petrel ( *Bulweria bulwerii* ) and the Mediterranean storm petrel ( *Hydrobates pelagicus* ) focus their flight and foraging effort at night (Dias et al. 2016; De Pascalis et al. 2025). A nocturnal lifestyle may help avoid daytime predators (at the colony) and exploit mesopelagic prey that migrate to the surface at night (Mougeot and Bretagnolle 2000). Despite their varied foraging ecologies, the activity patterns and feeding strategies of smaller petrels remain poorly understood (Rodríguez et al. 2019) compared to those of larger oceanic seabirds. Historically, our understanding of petrels' feeding behaviour had relied on stomach content and regurgitate analyses (Klages and Cooper 1997; Spear et al. 2007; Bester et al. 2011), with the well‐studied diel vertical migration of potential prey species being used as proxies for species‐specific foraging strategies and ecological roles in pelagic food webs (Murphy 1936; Imber 1973).

Gadfly petrels are oceanic seabirds of the genus *Pterodroma*, morphologically and anatomically adapted to exploit wind energy to perform prolonged and efficient dynamic soaring flights (Ventura et al. 2020) during which they mostly glide over the ocean surface with limited flapping‐flight (Warham 1977). Species in this group share similar ecological traits, primarily consuming deep sea mesopelagic prey (Cherel and Bocher 2022; Campioni et al. 2023), and employing a range of different feeding techniques (Imber 1973). Indeed, *Pterodroma* petrels are believed to forage in flight by contact ‘dipping’ to the surface (also known as ‘stooping’ or ‘aerial dipping’, defined as catching a prey in flight barely touching the sea surface, Ashmole and Ashmole 1967). They may also feed while resting on the water through surface‐seizing or scavenging, and less frequently, by searching for prey underwater (Haney 1986, 1987). Their specialisation in mesopelagic prey, including fish (mostly Myctophids) and cephalopods (Cherel and Bocher 2022), which ascend to the surface at night (Marohn et al. 2021), suggests a increase in *Pterodroma* petrels nocturnal foraging activity (Imber 1973; Bester et al. 2011; Cherel and Bocher 2022). Notably, unlike shearwaters or diving petrels, they are not regarded as proficient divers (Ashmole and Ashmole 1967; Warham 1977; Haney 1987; Bocher et al. 2000; Rayner et al. 2008; Dunphy et al. 2015; see references in Shoji et al. 2016), although their true diving capacity remains largely understudied.

We studied the at‐sea behaviour of the endangered Bermuda petrel ( *Pterodroma cahow* ), a gadfly petrel endemic to the western North Atlantic that breeds exclusively in the Bermuda Islands (Madeiros et al. 2012; Birdlife International 2018). To date, only two studies have examined the foraging ecology of this species (Raine et al. 2021; Campioni et al. 2023). Campioni et al. (2023) and showed that wind selection modulates petrel flight patterns, similar to its congeners, can be modulated through wind selection, likely facilitating extended and prolonged foraging trips while minimising energy expenditure. Although the diet of the Bermuda petrel is dominated by meso‐ and bathypelagic prey (primarily Myctophids fishes and cephalopods, Campioni et al. 2023), which would indicate substantial nocturnal foraging activity, previous GPS‐data analyses indicated similar proportion of ‘searching’ (i.e., putative foraging) and ‘transit’ behaviours during both day and night (Raine et al. 2021, Campioni et al. 2023). To better understand the species' daily activity budget, we combined the use of GPS, tri‐axial accelerometer, and time–pressure sensors to (i) characterise petrel diving behaviour, (ii) identify behavioural modes during flight and on water, and (iii) test for a diel‐based variation in these behaviours.

## 2 Material and Methods

### 2.1 Biologging Data Collection

Biologging devices were deployed on 25 incubating Bermuda petrels at Nonsuch Island, Bermuda (32°20′N, 64°40′W) between 22 January and 9 February 2023. Birds were temporarily equipped with biologgers attached to the back feathers or central tail feathers using Tesa tape 4651. Of the 25 deployed tags, 19 (76%) were successfully retrieved within 13–26 days (see Table 1 for tracking duration and other details). Three logger types were used in this study: (1) Axy5 loggers (Technosmart, Italy, 2.5 g) that included tri‐axial accelerometers (25 Hz), tri‐axial magnetometers (1 Hz), and time–pressure sensors (1 Hz) were deployed on the backs of 9 petrels; (2) AxyTrek loggers (Technosmart, Italy, 5 g) that included GPS sensors (1 fix every 5 min), tri‐axial accelerometers (25 Hz), and time–pressure sensors (1 Hz) were deployed on the backs of 5 petrels; and (3) Nano‐fix GEO loggers (PathTrack, United Kingdom, 3.4 g) that only recorded GPS locations (1 fix every hour) were deployed on the tail of 11 petrels (for details on attachment method see Campioni et al. 2023). Devices were deployed on breeding adults that weighed between 300 and 385 g (*N* = 25); thus, the heavier loggers (AxyTrek) corresponded to 1.3%–1.7% of body mass while the lighter loggers (Axy5) corresponded to 0.8%–0.65% of body mass. Due to weight restrictions, the three types of devices were deployed on different individuals. This choice was based on the need to balance high‐resolution data collection with minimal impact on the birds considering the species' critical conservation status. Furthermore, no detrimental effects were recorded on petrels tracked with biologgers (Campioni et al. 2023). Data from deployments and biologgers are included in Table 1 (see also Becciu et al. 2025).

**TABLE 1:** Summary of foraging trip metrics of Bermuda petrels tracked from Nonsuch Island in 2023.

Colony	Tag type	Nest	Bird	Sex	Total track			Foraging trip					
					Start	End	Duration (days)	Trip	Start	End	Duration (days)	Maximum distance (km)	Notes
A	Axy5	R819	E0368	M	2023‐01‐25 20:27	2023‐02‐03 1:03	8.2	1	2023‐01‐26 9:20	2023‐01‐31 1:35	4.7	NA	Complete
	Pathtrack	R820	E0487	F	2023‐02‐02 2:07	2023‐02‐17 18:07	15.7	1	2023‐02‐08 2:07	2023‐02‐17 6:07	9.2	1493	Complete
	Pathtrack		E0243	M	2023‐01‐23 2:03	2023‐02‐08 3:04	16.0	1	2023‐01‐26 11:03	2023‐02‐07 22:04	12.5	1503	Complete
	Axy5	R821	E0362	M	2023‐01‐23 0:00	2023‐01‐31 3:28	8.1	1	2023‐01‐24 6:20	2023‐01‐27 3:35	2.9	NA	Complete
	Pathtrack	R834	E0161	F	2023‐01‐29 2:00	2023‐02‐12 19:00	14.7	1	2023‐02‐01 6:00	2023‐02‐11 2:00	9.8	1100	Complete
	Axy5		E0182	M	2023‐01‐25 21:07	2023‐02‐03 5:37	8.4					NA	In burrow
	AxyTrek	R835	E0220	M	2023‐01‐26 0:00	2023‐02‐02 8:03	7.3	1	2023‐02‐01 7:35	2023‐02‐02 8:05	1.0	[549]	Incomplete
	Axy5		E0401	F	2023‐01‐25 20:15	2023‐02‐03 3:52	8.3					NA	In burrow
	AxyTrek	R836	E0171	M	2023‐02‐09 0:00	2023‐02‐18 22:01	9.9	1	2023‐02‐18 2:55	2023‐02‐18 22:00	0.8	[1075]	Incomplete
	Pathtrack	R837	E0801	F	2023‐01‐29 2:02	2023‐04‐01 14:06	62.5	1	2023‐01‐30 8:02	2023‐02‐01 6:02	1.9	232	Complete
								2	2023‐02‐11 7:02	2023‐02‐23 23:05	12.7	1433	Complete
	PathTrack	R839	E0363	F	2023‐01‐26 2:03	2023‐02‐19 19:05	24.7	1	2023‐02‐04 5:02	2023‐02‐18 2:05	13.9	1003	Complete
	Pathtrack	R840	E0484	M	2023‐01‐26 2:02	2023‐02‐08 3:02	13.0	1	2023‐01‐28 5:01	2023‐02‐03 3:02	5.9	675	Complete
B	AxyTrek	B12	C0888	F	2023‐02‐10 0:00	2023‐02‐18 7:33	8.3	1	2023‐02‐18 1:55	2023‐02‐18 7:35	0.2	[231]	Incomplete
	Axy5		E0252	M	2023‐01‐26 21:10	2023‐02‐04 4:47	8.3					NA	In burrow
	Axy5	B2	C0901	M	2023‐01‐26 20:00	2023‐02‐04 4:04	8.3	1	2023‐01‐27 6:35	2023‐02‐03 4:05	6.9	NA	Complete
	Axy5	B8	E0083	F	2023‐02‐10 0:00	2023‐02‐18 2:18	8.1	1	2023‐02‐10 0:00	2023‐02‐18 2:20	8.1	NA	Incomplete
	Axy5		C1036	M	2023‐01‐26 21:00	2023‐02‐03 17:59	7.9	1	2023‐01‐28 7:15	2023‐02‐03 18:00	6.4	NA	Incomplete
	Pathtrack	B9	E0552	F	2023‐01‐27 2:00	2023‐02‐14 23:00	18.9	1	2023‐02‐07 1:00	2023‐02‐14 23:00	7.9	1241	Complete

### 2.2 Defining Foraging Trips and At‐Sea Distribution Using Data From Birds Tagged With GPS‐Loggers

Foraging trips were defined as any period between a bird's departure and subsequent return to the colony, excluding locations within a range of 1 km from the colony thus removing positions on land and those possibly related to birds engaged in rafting behaviour in the proximity of the colony (Granadeiro et al. 2018). Only tracks with at least one complete foraging trip were used to calculate the maximum distance from the colony and the overall temporal duration of the trip.

A continuous‐time movement model was fitted to compute population‐level utilisation distribution (UD) of foraging areas using 90%, 75%, 50% and 25% autocorrelated kernel density estimates (AKDEs) for all the individuals using the functions akde from the package ‘ctmm’ (Fleming and Calabrese 2017; Silva et al. 2022). Specifically, the AKDEs were computed for each individual, and the population‐level UD was then obtained by combining these individual AKDEs using the *pkde* function with ‘weights = TRUE’, so that the aggregated UD accurately reflects the relative contribution of each individual, accounting for differences in track length. We fitted the AKDE using all tracks excluding locations within 50 km of the colony to ensure that only trips with unequivocal foraging behaviour (i.e., long foraging trips) were included in the analysis (for more details see Campioni et al. 2023).

### 2.3 Diving Activity

Depth data were collected at 1 Hz with a resolution of 0.005 m (Becciu et al. 2025). Two outliers with depths greater than 15 m were removed from the dataset, and these missing values were replaced with linearly interpolated values. These extreme observations were treated as depth sensor anomalies because they were isolated events that would have required traveling at vertical speeds > 15 m/s. A zero‐offset correction (ZOC) was used to correct for temporal variation in the accuracy of the depth sensor measurements (Luque and Fried 2011). We calculated the ZOC as the 10th percentile of depth over a 10‐min moving window. ZOCs ranged from 0 to 0.49 m. The ZOC was then subtracted from all depth measurements, and corrected values < 0 m were fixed at 0 m. We examined histograms of depth measurements binned to 0.1 m intervals while birds were engaged in foraging trips (see below for details of behavioural classification) for evidence of diving activity. We used a threshold of 0.5 m to define dives.

### 2.4 Accelerometer Behavioural Classification

We used tri‐axial accelerometers to collect high‐resolution activity data (Shepard, Wilson, Quintana, et al. 2008a; Nathan et al. 2012; Brown et al. 2013; Patterson et al. 2019; Wilson et al. 2020) from Bermuda petrels. Accelerometers record changes in acceleration on three planes: surge acceleration (forward‐backward), sway (left–right) and heave acceleration (up‐down). Behavioral patterns can often be readily identified by an observer looking at plots of different measures of acceleration (Shepard, Wilson, Quintana, et al. 2008a; Gómez‐Laich et al. 2009). However, for individuals tracked for multiple days at high resolution (in our case at 25 Hz) manual classification of entire tracks is impossible. We therefore used a supervised classification to assign behavioral states to the accelerometer data using the following workflow (followed by a detailed description of each step):

1. We calculated multiple metrics from the accelerometers that have been used to classify behaviors in other wildlife studies.
2. We manually classified a small subset of each individual's tracks to nine behavioural states.
3. We trained a random forest classification model using 50% of the manually classified data.
4. We validated the performance of the random forest classification model using the withheld classifications.
5. Predicted behavioural states for the entire accelerometer dataset using the classification model.

All accelerometer analysis was performed in R, version 4.3.2 (R Core Team 2023).

### 2.5 Accelerometer‐Derived Metrics

We used custom R scripts to calculate a range of accelerometer‐derived metrics shown to be useful in characterising bird behaviour and different modes of flight (Shepard, Wilson, Quintana, et al. 2008a; Williams et al. 2015; Patterson et al. 2019; Conners et al. 2021) including, surge acceleration, sway acceleration, heave acceleration, dynamic surge acceleration, dynamic sway acceleration, dynamic heave acceleration, body pitch, body roll, vectorial static body acceleration (VeSBA), vectorial dynamic body acceleration (VeDBA) and wingbeats. Vectorial dynamic body acceleration (VeDBA) and vectorial static body acceleration (VeSBA) were calculated following Williams et al. (2015). VeSBA measures gravitational acceleration which should be close to 1 when the animal is stationary or making linear movements, and greater than 1 when the bird is turning (Williams et al. 2015). VeDBA measures total dynamic movement of the animal, which can be influenced by intrinsic movements and environmental conditions (Williams et al. 2015). All measures except wingbeats were calculated over a 1‐s moving window. Wingbeats were identified using the ‘findpeaks’ function in the *pracma* package (version 2.4.2, Borchers 2022). Potential wingbeats were first identified as peaks in heave acceleration with an amplitude > 1 g where at least 2 successive peaks occurred within 4 Hz, these thresholds were identified throughout visual examination of heave acceleration.

### 2.6 Manual Behavioural Classification

**FIGURE 1:**
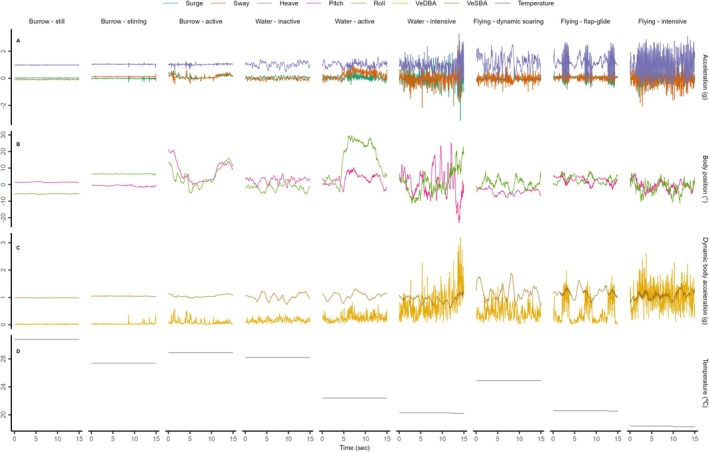
Examples of accelerometer‐derived metrics for nine behaviour classifications associated with the burrow, flying, and swimming. Each column shows 15‐s of behaviour for (A) raw acceleration in three axes (surge, sway, and heave), (B) body position (pitch and roll), (C) dynamic body acceleration (VeDBA and VeSBA), and (D) temperature.

We visually examined plots of the accelerometer‐derived metrics at different time scales (e.g., 15 s, 1 min, 5 min, 1 h) to identify consistent patterns that were likely associated with nine general behaviours: three behaviours in the burrow, three behaviours associated with swimming, and three flight behaviours. We expected that VeDBA would be broadly useful in distinguishing high activity behaviours from lower activity behaviours (Shepard, Wilson, Halsey, et al. 2008b); pitch and roll would indicate changes in posture (Shepard, Wilson, Quintana, et al. 2008a); heave acceleration, wing beats, and VeSBA would indicate flight behaviours (Conners et al. 2021; Gómez‐Laich et al. 2009; Patterson et al. 2019; Williams et al. 2015); and temperature would help identify periods in the burrow. From visual examination of the accelerometer‐derived measures (see section above), we found that acceleration in the heave axis, pitch, roll, VeSBA, VeDBA, and temperature were most useful in visually identifying characteristic behaviours that are likely associated with the burrow, flying and swimming behaviours. Table 2 provides a description of the characteristics of the nine behaviour classes, and Figure 1 shows examples of typical acceleration profiles for the behaviour classes. We randomly selected 400 15‐s segments from each bird tracked with an accelerometer (*n* = 4400 segments, representing 0.8% of all segments). Each segment was manually classified to one of nine possible behaviours by a single observer (AP). Segments were each classified twice in random order. Segments that were not classified consistently to the same behaviour were reviewed a third time to determine a final classification.

**TABLE 2:** Description of the movement characteristics used to manually classify training segments to nine behaviour classes.

Behaviour	Behaviour characteristics	Training segments
Burrow—still	Consistent high temperature; low VeDBA; virtually no variation in surge, sway, and heave axes	1350
Burrow—stirring	Consistent high temperature; low VeDBA with max VeDBA < 1 g; small amplitude variations in surge, sway, and heave axes	715
Burrow—active	Consistent high temperature; max VeDBA > 1 g; sustained variation in VeDBA, surge, sway, and heave axes; changes in pitch and roll greater than 10°	338
Flying—dynamic soaring	Consistent fluctuations in VeSBA with peaks > 1.5 g; consistent low frequency fluctuations in heave acceleration with peaks > 2 g; absence of wing beats	102
Flying—flap‐glide	Consistent fluctuations in VeSBA with peaks > 1.5 g; consistent fluctuations in heave acceleration with peaks > 2 g; at least one bout of 3 successive wing beats	540
Flying—intensive	Fluctuations in VeSBA with peaks > 1.5 g; luctuations in heave acceleration with peaks > 2 g; sustained bouts of wing beats > 4 s, unusually high amplitude wingbeats (> 2 g); irregular variation in pith or roll	70
Water—inactive	Low mean and peak VeDBA; random low amplitude variation in VeDBA, surge, sway, and heave acceleration; minimal variation in VeSBA	88
Water—active	Mean VeDBA > 0.1 g and < 1 g; non‐random variation in surge, sway, and heave acceleration; fluctuations in pitch or roll > 10° and < 50°; minimal variation in VeSBA	130
Water—intensive	Low variation in VeSBA, peak VeSBA < 1.5 g; high mean VeDBA > 1 g, peak VeDBA > 2 g; large variation in pitch/roll > 50°; intense non‐random variation in surge, sway, and heave acceleration	116

*Note:* Final column shows the number of randomly selected segments classified to each behaviour. Although 4400 random segments were initially selected, 26 segments could not be reliably assigned to a behaviour class and were removed from the training dataset.

### 2.7 Random Forest Model Training, Evaluation and Behavioural Prediction

Manually classified data were split into training (50%) and validation (50%) data. For each 15‐s segment in the tracking data, we calculated summary statistics of the 12 metrics described in Section ‘*Accelerometer‐derived metrics*’. Summary statistics included mean, inter‐quartile range (IQR), 10th quantile, 90th quantile and sum (only applied to wingbeats); a detailed explanation of how each metric was summarized is provided in Table S1. This resulted in 36 potential predictor variables for behavioral classification.

We used the training data to fit a classification algorithm for the nine behavioural states. Behavioural classification was conducted using a random forest classification model with the *ranger* package (version 0.16.0, Wright and Ziegler 2017). Model training and variable selection was done using the *caret* package (version 6.0–94, Kuhn et al. 2021). The ranger model has three parameters (minimum node size, mtry, and extratrees) that need to be optimised. This model tuning was done using repeated k‐fold cross validation with 5 repeats of 10 folds, where within each fold, 50% of the data were used in model training and 50% were withheld for model testing. Behavioural classes were up‐sampled (e.g., observations of each behaviour were resampled with replacement) to ensure even sample sizes across all behaviours within the training dataset. Model tuning parameters were selected using the random grid search option with 18 different combinations of values for the hyper‐parameters of minimum node size (min.node.size), number of variables used at each node (mtry), and splitting rule (gini or extratrees). We used Recursive Feature Elimination (RFE) to reduce the number predictor variables in the model, using the ‘*rfe’* function in the caret package (Kuhn et al. 2021). This algorithm fit a full model with all possible predictors, then recursively removed predictors based on the variable importance ranking and refits the reduced models. Kappa was compared across all the models; the variables included in the simplest model within 1% of the highest accuracy were used in the final model. The final model was fit using 14 predictor variables (Table S1) with min.node.size = 3, mtry = 11, and split rule = extratrees.

We used the final random forest model to predict behavioral classifications for all segments. The withheld validation segments were used to estimate the accuracy of the final model on independent data. We assessed overall model performance based on the overall classification accuracy and Kappa. We explored the behavior‐specific classification accuracy by looking at balanced accuracy for each of the nine behaviors and for the three main behavioral modes combined (burrow, water, flying). We looked at the variable importance to determine which accelerometer measures contributed the most to classification accuracy.

### 2.8 Activity Budget and Diel Patterns Relative to Sunlight

**FIGURE 2:**
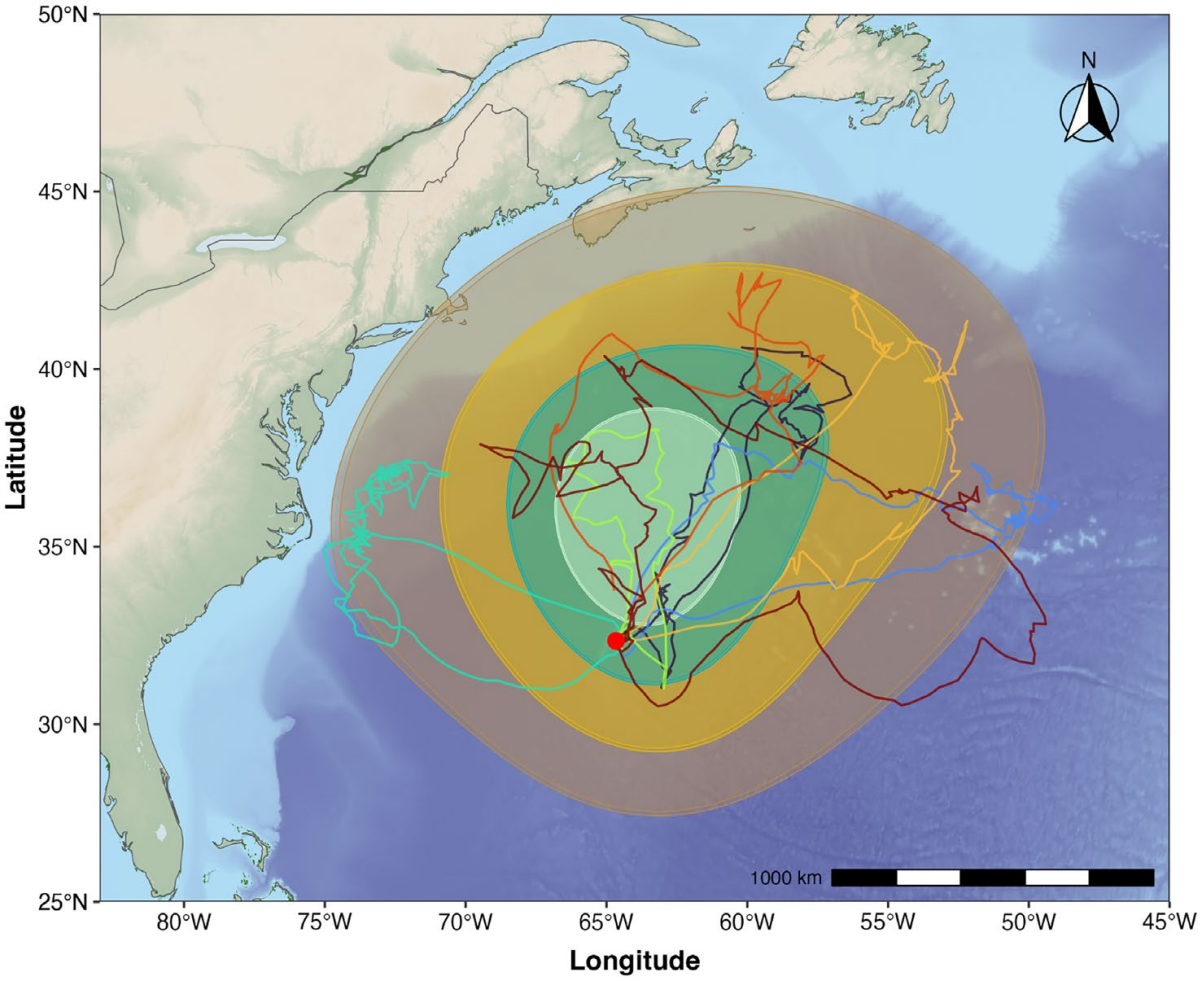
Complete foraging trips for seven Bermuda petrels tracked from Nonsuch Island, Bermuda (red point) during incubation in 2023 using PathTrack Nano‐Fix GPS units, and weighted Population‐Level UD 90 (brown), UD 75 (orange), 50 (green) and 25% (light green) calculated using all trips.

We calculated the proportion of time spent in the six at‐sea behaviours over 1‐h intervals (from 8 birds, hereafter accelerometer‐derived behaviours), and generated an activity budget for each individual. To assign light conditions to each time step, we estimated the sun angle (in radians) using the coordinates of the breeding colony (Thieurmel et al. 2019). Although individual birds were distributed across a broader spatial area at sea, we evaluated the variation in sun angle across the population‐level utilisation distribution (UD; Figure 2) and found that the maximum differences in sun angle at sunrise and sunset were negligible (0.0009 rad at sunrise; 0.001 rad at sunset; see Figures S3 and S4). This confirmed that using the colony coordinates to estimate sunrise and sunset times provided a good proxy for light conditions experienced across the distribution range.

To investigate how light conditions influenced both the probability of occurrence and the proportion of time petrels spent in each behaviour, we fitted zero‐inflated beta GLMMs in R (glmmTMB v1.1.8; Brooks et al. 2017), with sun angle as a fixed effect, bird identity as random intercepts and slopes, and a first‐order autoregressive error structure. We generated posterior predictive plots to check for systematic discrepancies between model output and the observed data using the ‘performance’ package in R (version 0.10.8, Lüdecke et al. 2021). Because there was large variation in the tracking duration among individuals we refit models excluding each individual and compared the resulting coefficients for fixed and zero‐inflation effects to confirm that model estimates were not highly influenced by any individual track.

## 3 Results

Data were obtained from 8 of 9 Axy5 deployments (89%), 3 of 5 AxyTrek deployments (60%), and 7 of 11 PathTrack deployments (63%). Trip duration from the Axy5 and AxyTrek devices averaged 8.2 and 8.5 days, respectively, although only three of the devices (all Axy5 units) recorded a complete foraging trip (Table 1). Three of the petrels with Axy5 devices remained in their burrow throughout the deployment. Biologgers with GPS sensors recorded eight complete foraging trips (from 7 Pathtrack units) and three incomplete trips (AxyTrek units).

### 3.1 Diving Behaviour

**FIGURE 3:**
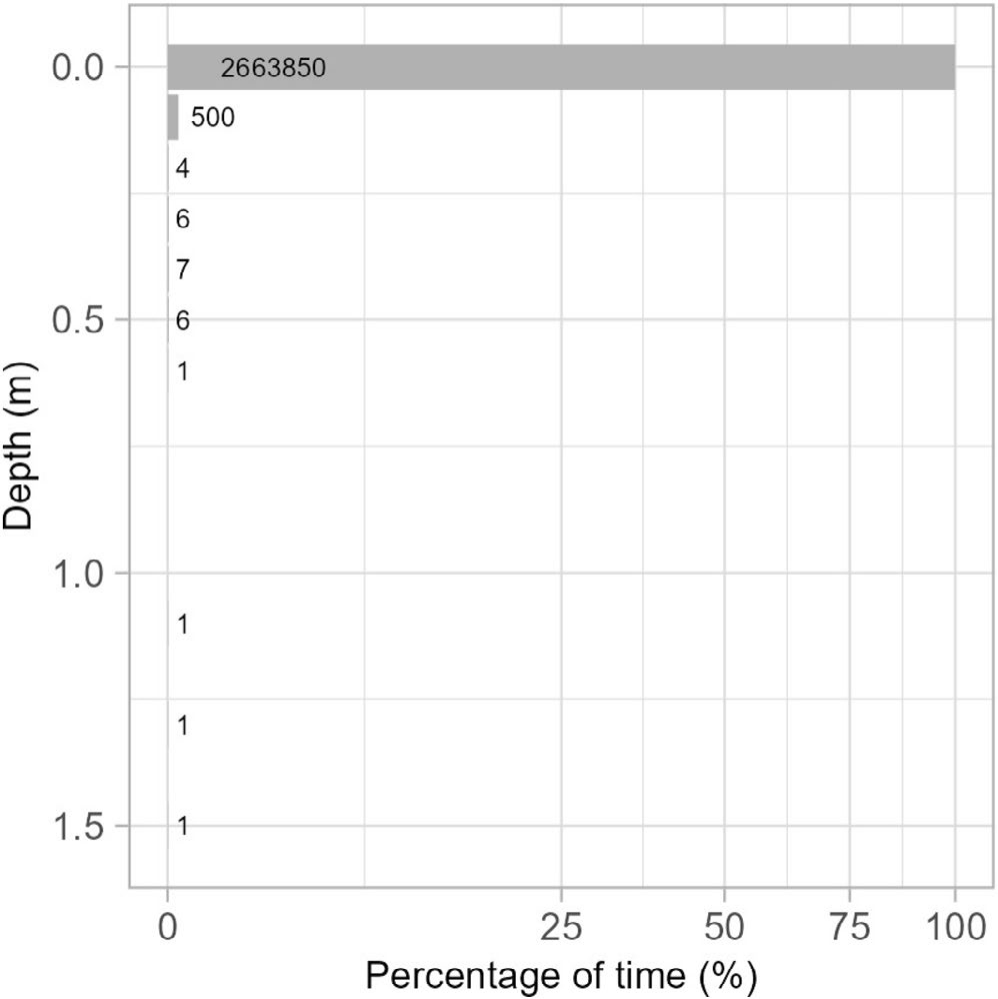
Percentage of time within 0.1 m depth classes, pooled across all foraging trips. Numbers to the right of bars represent the number of depth measurements in that depth class. The *x*‐axis is square‐root transformed to improve visualisation of the range of values in the plot.

We found evidence that petrels engaged in very limited diving activity. After applying the Zero‐offset correction (ZOC) to account for drift in the pressure sensor, less than 0.001% of depth measurements from 8 petrels exceeded 0.1 m, with the maximum depth recorded at 1.57 m (Figure 3). There were only five dives deeper than 0.5 m (median = 0.59 m). Four dives lasted only 1 s, while the deepest dive to 1.57 m lasted 4 s. For each dive, we examined the pitch profile for a sigmoidal pattern of rapidly declining and rising pitch, which was evident for all dives.

### 3.2 Accelerometer Behaviour Classification

Overall, the random forest model correctly classified behaviours in the testing data set with 87.1% accuracy (CI: 85.7–88.5) with a Kappa of 83.9%. Most individual behaviours had balanced classification accuracy between 85.6% and 96.5%, except for the ‘flying‐intensive’ behaviour (61.7%, Table 3). When behaviours were further collapsed into three main activity modes of burrow, water, and flying, model accuracy increased to 98.7% (CI: 98.2–99.1), with a Kappa of 97.7% and balanced accuracy for individual classes between 96.3% and 99.6%. This shows that most misclassifications occurred within these three main behavioural modes. This demonstrates that the random forest model could consistently apply the behavioural characteristics identified through manual classification to the entire dataset. The five most important variables in the classification were mean VeDBA (100%), the 90th quartile of VeDBA (82.3%), mean dynamic sway acceleration (78.8%), mean dynamic heave acceleration (76.1%) and the IQR of heave acceleration (74.4%, Figure S1).

**TABLE 3:** Behaviour specific performance metrics for the random forest model predicting Bermuda petrel behaviour from accelerometer data.

Behaviour	Precision	Recall	Accuracy	Mode	Precision	Recall	Accuracy
Water—inactive	0.875	0.714	0.856	Water	0.936	0.931	0.963
Water—active	0.736	0.877	0.933				
Water—intensive	0.867	0.788	0.892				
Flying—dynamic soaring	0.920	0.955	0.965	Flying	0.982	0.989	0.989
Flying—flap‐glide	0.868	0.890	0.936				
Flying—intensive	0.529	0.237	0.617				
Burrow—still	0.928	0.925	0.947	Burrow	0.998	0.994	0.996
Burrow—stirring	0.771	0.762	0.860				
Burrow—active	0.800	0.804	0.894				

*Note:* Left‐hand columns show results for detailed behavioural classes and right‐hand columns show results for general behavioural modes.

Burrow behaviours had low measures of activity, relative to the other behaviour classes, across all accelerometer metrics and relatively high mean temperatures (Figure S2). Burrow‐still had the lowest values across all measures of acceleration. Burrow‐stirring had higher activity measures than burrow‐still, but values were lower than for other behaviours. The burrow‐active behaviour had similar values for mean VeDBA and mean dynamic surge compared to the water‐inactive behaviour, however burrow‐active had lower IQR heave acceleration and lower IQR VeSBA (Figure S2).

The three water behaviours were distinguished from the three flying behaviours by lower IQR in both the heave axis and VeSBA (Figure S2). Water‐inactive had very low VeDBA and likely represented periods of resting on the water when movement was coming from the movement of the water rather than the motion of the bird. Water‐active had higher activity overall than water‐inactive but still had low VeDBA compared to other at‐sea behaviours (Figure S2). The water‐active class likely represents periods of minimal activity, for example when the bird was actively maintaining its position in the current through swimming. Water‐active could include passive foraging movements, like picking at prey at the surface, but not movements consistent with active pursuit of prey or competition with other birds. Water‐intensive had the highest activity measure of all the water behaviours, especially pronounced in mean VeDBA, wingbeats, and mean dynamic surge. High dynamic surge was likely associated with repeated dipping of the head into or towards the water, potentially to catch prey or as part of bathing/preening. The occurrence of sporadic wingbeats could also have been associated with preening or short hops across the water.

The three flight behaviours were all characterised by high values of mean VeDBA, IQR heave, and IQR VeSBA (Figure S2). Flap‐glide flight and dynamic soaring flight had similar values for mean VeDBA, but flap‐glide had higher numbers of wingbeats and lower IQR VeSBA. In dynamic soaring flight, the large variation in VeSBA is consistent with petrels using favourable wind conditions to reduce energetic costs. Intensive flight had more wingbeats per second than flap‐glide flight, as well as more dynamic acceleration in the surge axis. Higher wingbeats could be associated with take‐offs and landings or other aerial manoeuvring. Higher dynamic heave acceleration, similar to water‐intensive, could indicate dipping or plunging towards the water. The intensive flying behaviour had much lower accuracy against the validation data then the other behaviour classes, as it likely reflects a range of short‐duration behaviours that share the characteristics of unusually high wingbeats and high IQR in the VeSBA. Rather than attempting to split this class into multiple behaviours to attain higher accuracy, we chose to treat this classification as a catch‐all behaviour for highly intensive movements during flight. Further validation data through observations of tagged individuals would likely be necessary to tease out what petrels are doing within this behaviour. As *Pterodroma* petrels are known to forage in flight (Ashmole and Ashmole 1967; Murphy 1936), it is plausible that all three flight behaviours could include pursuit and capture of prey.

### 3.3 Activity Budgets

**FIGURE 4:**
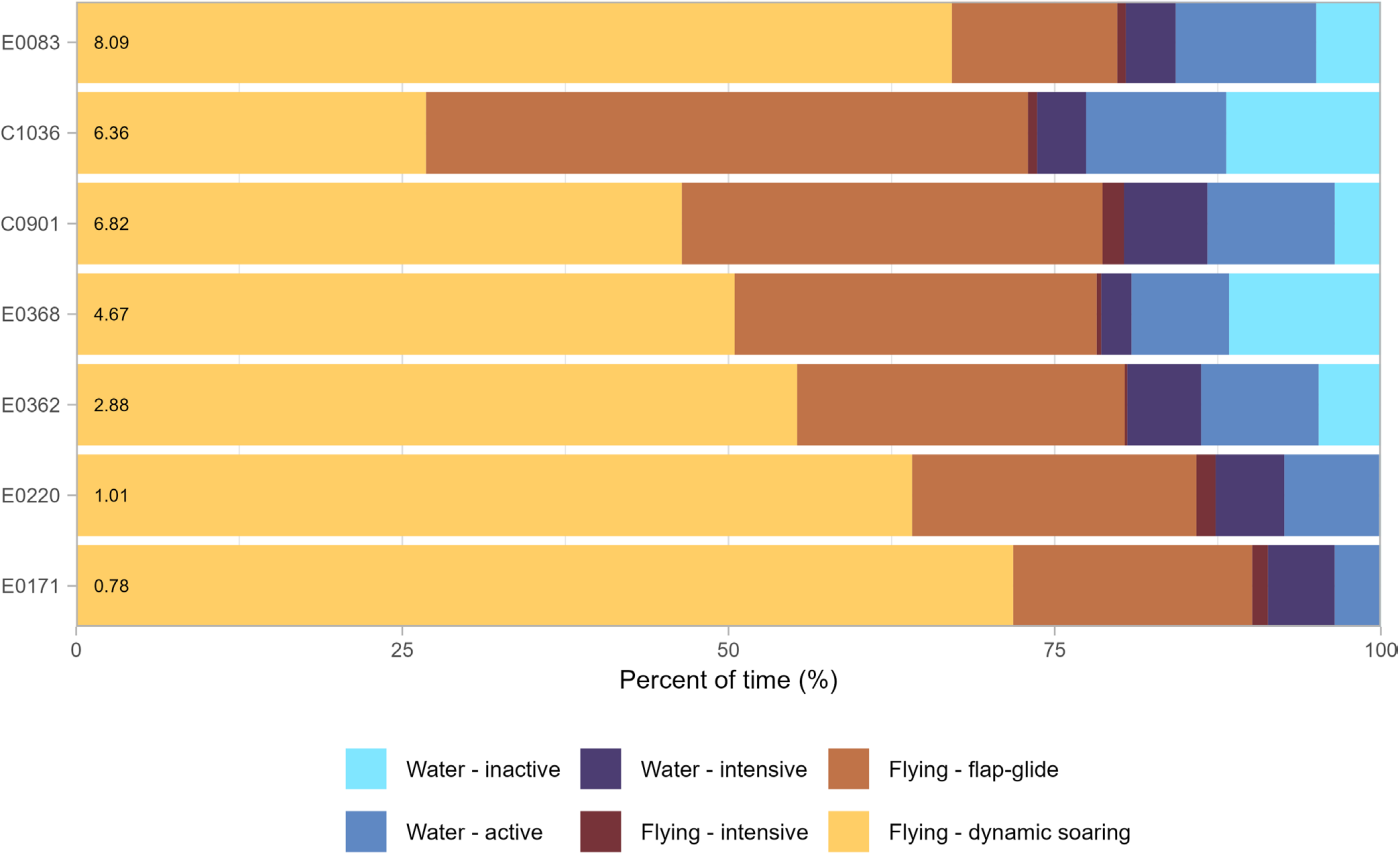
Activity budgets while at sea of the 7 Bermuda petrels tracked from Nonsuch Island with accelerometers in 2023. Colours indicate the six behaviours classified using a random forest model, and individual bird identities are indicated on the *y*‐axis, while tracking duration in days is shown to the right of bird identities. Note there was large variation in the length of times individuals tagged with accelerometers were tracked at sea (range: 0.2–8.1 days) and only 3 of 7 birds were tracked through an entire foraging trip (see Table 1).

**FIGURE 5:**
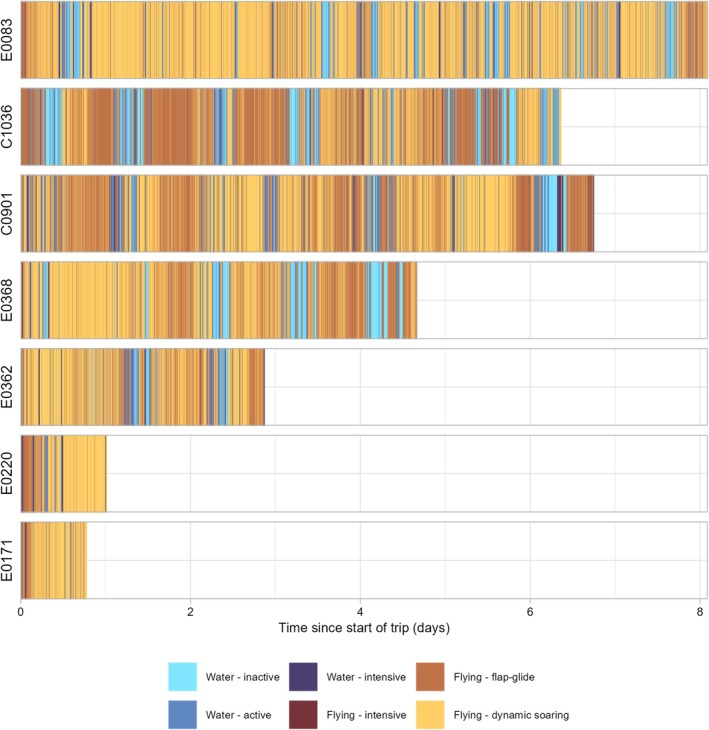
Sequence of classified behaviours within deployments of accelerometers on Bermuda petrels. Colours indicate the six behaviours classified using a random forest model; individual bird identities are indicated on the *y*‐axis. Note that dates are different along the *x*‐axis for each bird because deployments were not concurrent.

During foraging trips, petrels spent over 75% of their time in flight (Figures 4 and 5), primarily in dynamic soaring flight accounting for 52.9% ± 14.5% (range: 26.8%–67.1%) and flapping‐gliding flight 27.6% ± 11.2% (range: 12.7%–46.1%) (Figure 4). Across all birds, only a small fraction of time (0.9% ± 0.6%; 0.2%–1.7%) was classified as intensive flying. On the water, petrels spent similar proportions of time in active (9.2% ± 1.5%, range: 7.3%–10.8%) and inactive (6.2% ± 4.7%, range: 0.14%–11.9%) behaviours, with water‐intensive behaviour representing 4.5% ± 1.5% (range: 2.3%–6.4%). There was notable variation in time spent in certain activities among individuals. C1036 had a higher proportion of time in flap‐glide flight compared to other petrels (Figure 4). Birds tracked for shorter durations (E0220 and E0171) spent little time inactive on the water, which could indicate that petrels only rest after reaching profitable foraging grounds or foraging extensively although other petrels were inactive on the water within the first 24 h of their trips (Figure 5).

### 3.4 Diel Patterns Relative to Sunlight

**FIGURE 6:**
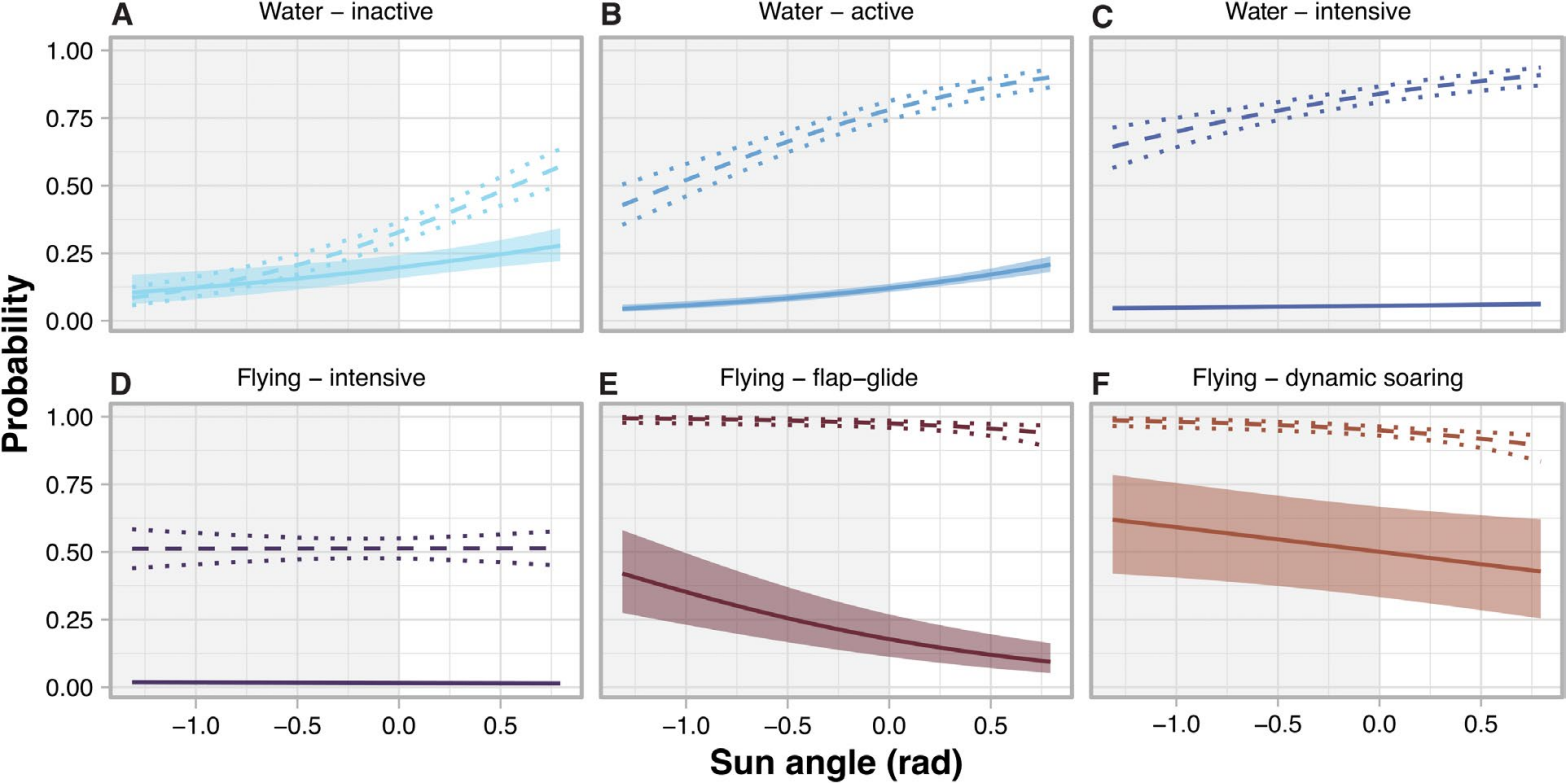
Predicted effect of sun angle on the time spent in different behaviours during foraging trips by incubating Bermuda petrels. Models were run using behavioural modes derived from accelerometer (A–F). Dashed lines show the probability of occurrence for each behaviour and dotted lines are 95% confidence intervals. Solid lines show the proportion of time petrels were predicted to engage in each behaviour conditional on the probability that the behaviour occurs (shaded areas are 95% confidence intervals). The grey‐white area of the plot represents the dark–light conditions.

Sun angle significantly affected the time allocation to all accelerometer‐derived behaviours except for intensive flying (Figure 6A–F and Table 4). Dynamic soaring flight was the most prevalent behaviour across all conditions. Both dynamic soaring and flap‐glide behaviours had a high probability of occurring at all sun angles, however their probabilities declined as sun angle increased (Figure 6E,F and Table 4). The proportion of time spent in flap‐glide flight behaviour had a strong negative association with sun angle (Figure 6E). In contrast, all water behaviours increased in both occurrence and duration with higher sun angles (Figure 6A–C, Table 3). Notably, water‐active and water‐intensive behaviours had higher probability of occurring during the day than the water‐inactive behaviour (Figure 6B,C), even though birds overall spent more time in water inactive than water‐active behaviour. This likely reflects that resting intervals occurred more sporadically but lasted longer than other water‐surface activities. No significant relationship was found between sun angle and intensive flying behaviour, which was the most uncommon behaviour across all conditions (Figure 6D). Re‐running models successively excluding individuals from analysis did not change the observed relationships between solar angle and behaviour. Collectively, the results indicate that Bermuda petrels are primarily aerial at night and only spend significant time on the water during daylight.

**TABLE 4:** Model coefficients for zero‐inflated beta generalised linear mixed model predicting the effect of sun angle on the probability of Bermuda petrels engaging in different behaviours during foraging trips (see Figure 6).

Behaviour	Zero‐inflated model		Conditional model	
	Intercept	Sun angle	Intercept	Sun angle
Water—inactive	0.72 ± 0.09***	−1.26 ± 0.14***	−1.40 ± 0.14***	0.57 ± 0.16***
Water—active	−1.3 ± 0.10***	−1.2 ± 0.14***	−1.99 ± 0.07***	0.82 ± 0.09***
Water—intensive	−1.66 ± 0.11***	−0.81 ± 0.15***	−2.84 ± 0.07***	0.14 ± 0.05**
Flying—intensive	−0.05 ± 0.08	−0.003 ± 0.11	−4.15 ± 0.06***	−0.12 ± 0.06
Flying—flap‐glide	−3.7 ± 0.26***	1.2 ± 0.45**	−1.53 ± 0.27***	−0.92 ± 0.17***
Flying—dynamic soaring	−2.9 ± 0.18***	1.0 ± 0.31***	0.003 ± 0.35	−0.39 ± 0.19

*Note:* Values are parameter estimates ± standard errors on the logit scale. *** is 0.001, ** is 0.01.

### 3.5 Foraging Trips and At‐Sea Distribution Calculated Using Loggers With GPS Sensor

Eight complete incubating foraging trips (seven long and one short, see Table 1) were recorded using GPS loggers (Pathtrack devices; Figure 6). The mean (± SD) trip duration was 10.3 ± 2.9 days, with birds reaching an average maximum distance of 1207 ± 305 km from the colony (Table 1). The at‐sea distribution encompassed 3,303,963 km^2^ (UD 90%), with a principal core area of 314,130 km^2^ (UD 25%) overlapping the high seas of the western North Atlantic (Figure 6).

## 4 Discussion

Our results indicate that Bermuda petrels are predominantly nocturnal surface feeders, rarely engaging in shallow diving during their extensive foraging trips in the open ocean. In our TDR dataset, 99.99% of depth readings were shallower than 0.1 m (Figure 3), and over 31 tracking days we recorded only five dives more shallow than 0.5 m—each lasting about 1 s. Thus, although Bermuda petrels can perform brief, shallow dives, such behaviour appears to be an exceedingly rare facet of their foraging strategy during the incubation period. Unlike many other Procellariiformes capable of prolonged deep dives (Rayner et al. 2008; Bester et al. 2011; see references in Shoji et al. 2016; Soldatini et al. 2023), *Pterodroma* petrels generally do not rely on diving to forage. However, unexpected deep dives have been observed in species poorly adapted for diving such as albatrosses (Bentley et al. 2021; Guilford et al. 2022), suggesting that even birds supremely adapted to long‐distance dynamic‐soaring flight may have the capacity to dive deeper under specific circumstances (Rayner et al. 2008; Guilford et al. 2022). Although most *Pterodroma* petrels perform shallow dives, rarely exceeding two meters, exceptions exist, such as the Cook's petrel and the Grey‐faced petrel, which can dive to approximately 27 and 23 m, respectively (Rayner et al. 2008; Taylor 2008). The absence of deep dives in Bermuda petrels implies that the capture of mesopelagic and bathypelagic prey should occur predominantly at night when these organisms are most accessible near the water surface.

Accelerometer data revealed that petrels spent over 75% of their time in flight, with dynamic‐soaring flight (averaging 53% of total activity) as the primary mode. This flight strategy, consistent with observations in species like Murphy's petrel (Clay et al. 2017), allows these birds and related species—such as petrels, albatrosses, and shearwaters—to traverse vast oceanic areas with minimal energy expenditure (Sachs et al. 2013; Shepard 2022). Consequently, dynamic soaring flight plays a crucial role in maximizing the foraging area and is likely modulated by wind conditions, as suggested by previous studies on *Pterodroma* petrels (Ventura et al. 2020; Halpin et al. 2022; Campioni et al. 2023). Although our analysis did not include environmental variables, due to the absence of GPS data in most of the biologgers used, there is strong evidence that petrels actively select favorable wind directions to support their extensive movements (Campioni et al. 2023) and enhance foraging success (Ventura et al. 2020, 2022).

Bermuda petrels also showed clear variation in diel activity they were less active and spent more time on the water during the day, with all three water behaviours increasing at higher sun elevations (Figure 6). This diurnal resting activity mirrors that observed in Stejneger's petrels ( *Pterodroma longirostris* , Clay and Brooke 2024), Trindade petrels ( *Pterodroma arminjoniana* , Krüger et al. 2016) and Desertas petrels ( *Pterodroma deserta* , Ramirez et al. 2013), contrasting with patterns of Pacific gadfly petrels, like the Chatham petrel ( *Pterodroma axillaris* ) and the Murphy's petrel, which do not exhibit such pronounced day‐night differences (Rayner et al. 2012; Clay et al. 2017, 2019). The water‐intensive behaviour was the least frequent of the water behaviours we observed for Bermuda petrel, characterised by bursts of ‘hyperactivity’, which may indicate scavenging or prey seizing, a feeding technique potentially linked to the ingestion of large squids whose DNA is often found in petrels' stomach content (Ashmole and Ashmole 1967; Klages and Cooper 1997; Bester et al. 2011) or of big chunks of fishes found in Bermuda petrel nests (Campioni L. personal observations). However, without external validation, we cannot conclusively attribute this water‐intensive behaviour to surface foraging. In addition, we acknowledge that a certain degree of interindividual variability in activity budget might result from the limited number of individuals, and for some of them a limited tracking duration.

How Bermuda petrel access deep‐sea prey remains unclear. Although, some mesopelagic fishes, such as lanternfish, have epipelagic larvae (0–20 m depth Namiki et al. 2017) and many squid species, including those known to be part of the Bermuda petrel's diet (Campioni et al. 2023), undergo a planktonic embryo stage (*Stigmatoteuthis hoylei* and *Histioteuthis corona* in https://www.sealifebase.ca/search.php), these factors alone do not fully explain the presence of adult mesopelagic fishes in diet analyses of *Pterodroma* petrels (Silva M unpublished data). One possibility is that interaction with demersal or deep‐sea cephalopod fisheries, provide additional food sources through scavenging on discards (Votier et al. 2023). Further research examining the interplay between prey ecology and oceanographic features (Godø et al. 2012) is warranted to shed light on this complex predator–prey dynamic.

Bermuda petrel activity fluctuated over the diel cycle. Flight behaviors increased with negative sun elevation values, indicating heightened nocturnal activity. All three flight behaviors might include aerial dipping—a foraging technique previously described in *Pterodroma* petrels (Murphy 1936; Ashmole and Ashmole 1967)—which could explain how the birds capture deep‐sea prey during the diel vertical migration (Marohn et al. 2021). Although factors such as breeding stage and moon illumination are known to affect birds' nocturnality (Rayner et al. 2012; Clay et al. 2017; Pastor‐Prieto et al. 2019; Bonnet‐Lebrun et al. 2021), our limited tracking period precluded a detailed analysis of moonlight and breeding stage effects. Nevertheless, as observed in other *Pterodroma* petrels (Ventura et al. 2024), breeding duties likely override moonlight influence on at‐sea behavior during the breeding season.

As a first attempt at measuring fine‐scale foraging behaviour of this small, highly pelagic, endangered seabird species, our study provides important insights into how to improve efforts to advance our understanding of the ecology and conservation of similar species. *Pterodroma* petrels present a challenge for biologging studies because their small body size limits the size of loggers that can be used, while their long foraging trips necessitate extended tracking durations. Unfortunately, we did not obtain significant data from loggers that combined GPS with TDR‐accelerometry (AxyTrek loggers), because we used a higher GPS fix rate (5 min) that quickly drained the battery. We suggest future studies use loggers with all three sensors but reduce the GPS fix rate to extend battery life. Ideally, loggers that combine GPS tracking with accelerometery and magnetometry could be used to dead‐reckon spatial tracks between GPS positions (Gunner et al. 2021a, 2021b). The data collected here from Axy5 loggers included magnetometer tracking that could be interrogated to better understand how birds are using changes in heading during dynamic soaring, but unfortunately these data do not have paired GPS positions to compare movements to wind conditions or recreate spatial movements. Finally, our study had a limited sample size of accelerometer data over complete foraging trips and there was notable variation in behaviour among individuals and throughout foraging trips. A larger sample size of complete trips and potentially repeat trips would be needed to fully characterise the foraging behaviour and variability within this species.

The Bermuda petrel is highly active with individuals spending 80% of their time in flight while foraging over vast oceanic areas. As such, large‐scale ecosystem changes, such as increasing SST and marine heatwaves (Furness 2016), alteration in the Gulf stream, or changes in wind regimes (Schreiber 2001) could disrupt the spatiotemporal availability of prey and the energetic balance of this aerial forager. Furthermore, its pronounced nocturnality may increase the vulnerability to offshore artificial lights, particularly those from marine traffic, which can attract and disorient birds (Dierschke et al. 2016). Further research should address inter‐annual variation in foraging distribution, environmental influence on movement patterns and potential shifts in at‐sea habitat use in response to climate‐induced changes in oceanography and human activities.

## Author Contributions

**Paolo Becciu:** conceptualization (equal), formal analysis (equal), methodology (equal), writing – original draft (equal). **Allison Patterson:** data curation (equal), formal analysis (equal), methodology (equal), writing – original draft (equal). **Carina Gjerdrum:** funding acquisition (equal), investigation (equal), resources (equal), validation (equal), writing – review and editing (equal). **Jeremy Madeiros:** investigation (equal), resources (equal), writing – review and editing (equal). **Letizia Campioni:** conceptualization (equal), funding acquisition (equal), investigation (equal), project administration (equal), validation (equal), writing – original draft (equal).

## Conflicts of Interest

The authors declare no conflicts of interest.

## Supporting information


**Data S1.** Supporting Information.

## Data Availability

Data are available here: https://doi.org/10.5281/zenodo.15364504.
