# Supplementary material for: Fine‐Scale Movement Data Reveal Primarily Surface Foraging and Nocturnal Flight Activity in the Endangered Bermuda Petrel
Source: Ecol Evol. 2025 Jun 30;15(7):e71647. doi: 10.1002/ece3.71647 (PMC12207655; doi:10.1002/ece3.71647)
Supplement: Supplementary file 1 — Data S1. Supporting Information. [file ECE3-15-e71647-s001.docx]

**Supplemental Information for:**

**Fine-scale movement data reveal primarily surface foraging and nocturnal flight activity in the endangered Bermuda petrel**

**Table of Contents:**

| **Table S1** | Page 2 |
| --- | --- |
| **Figure S1** | Page 3 |
| **Figure S2** | Page 4 |
| **Figure S3** | Page 5 |
| **Figure S4** | Page 6 |

**Table S1.** Description of the 36 accelerometer derived statistics used to summarize behaviour characteristics within each 15-sec segment of tracks. The last column indicates which variables were included in the final model after recursive feature elimination.

| **Accelerometer measure** | **Summary statistic** | **Included in final model** |
| --- | --- | --- |
| Temperature (C) | Mean | Y |
| Surge (g) | Mean |  |
|  | 10th quartile |  |
|  | 90th quartile |  |
|  | Inter-quartile range |  |
| Sway (g) | Mean |  |
|  | 10th quartile |  |
|  | 90th quartile |  |
|  | Inter-quartile range |  |
| Heave (g) | Mean |  |
|  | 10th quartile |  |
|  | 90th quartile | Y |
|  | Inter-quartile range | Y |
| Wing beats | Sum | Y |
| VeDBA (g) | Mean | Y |
|  | 10th quartile |  |
|  | 90th quartile | Y |
|  | Inter-quartile range |  |
| VeSBA (g) | Mean |  |
|  | 10th quartile |  |
|  | 90th quartile |  |
|  | Inter-quartile range | Y |
| Pitch | Inter-quartile range |  |
| Roll | Inter-quartile range |  |
| Dynamic heave (g) | Mean | Y |
|  | 10th quartile |  |
|  | 90th quartile | Y |
|  | Inter-quartile range |  |
| Dynamic sway (g) | Mean | Y |
|  | 10th quartile |  |
|  | 90th quartile | Y |
|  | Inter-quartile range |  |
| Dynamic surge (g) | Mean | Y |
|  | 10th quartile | Y |
|  | 90th quartile |  |
|  | Inter-quartile range | Y |


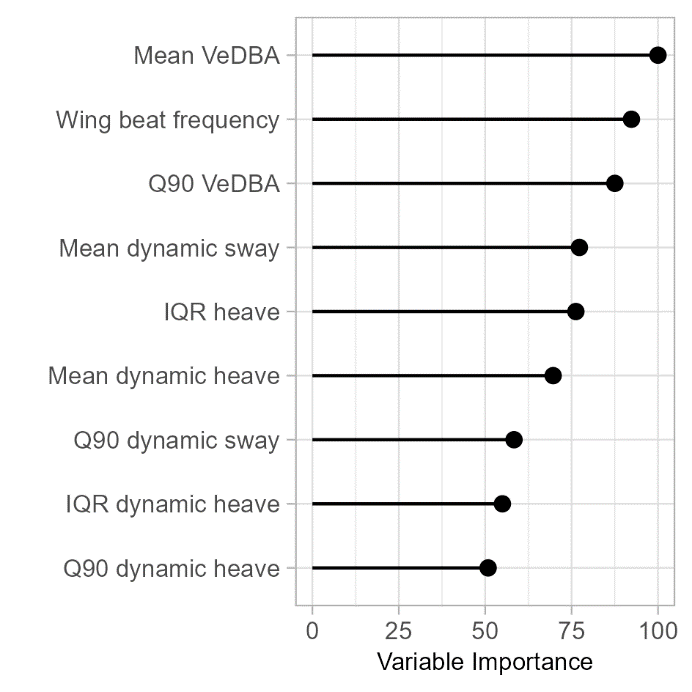


**Figure S1.** Relative variable importance for the final random forest model classifying behavioural states of Bermuda petrels from accelerometer tracks.


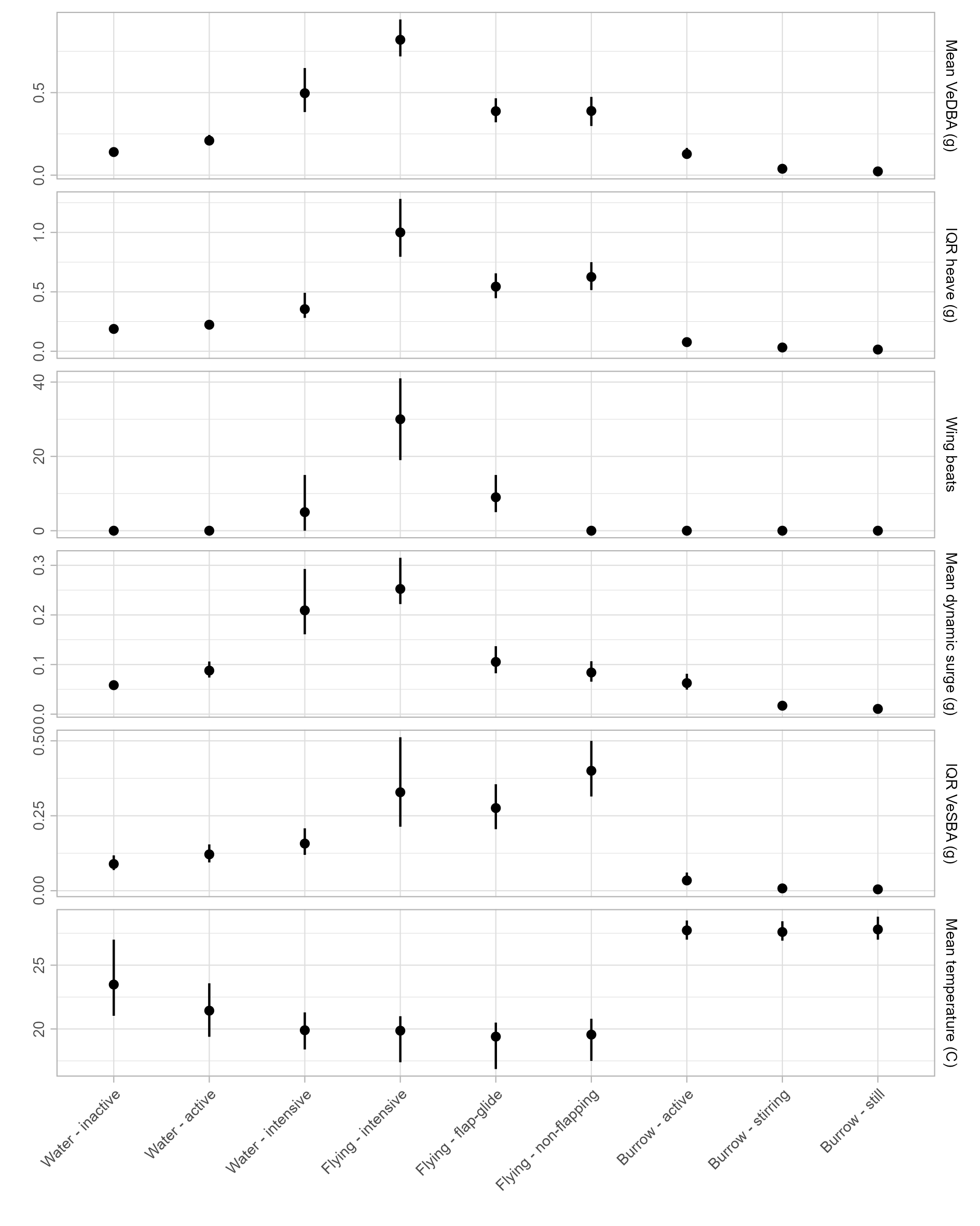


**Figure S2.** Distribution of acceleration metrics within each behaviour. Points show median value of all segments classified to a behaviour and error bars show 25^th^ – 75^th^ quantiles.


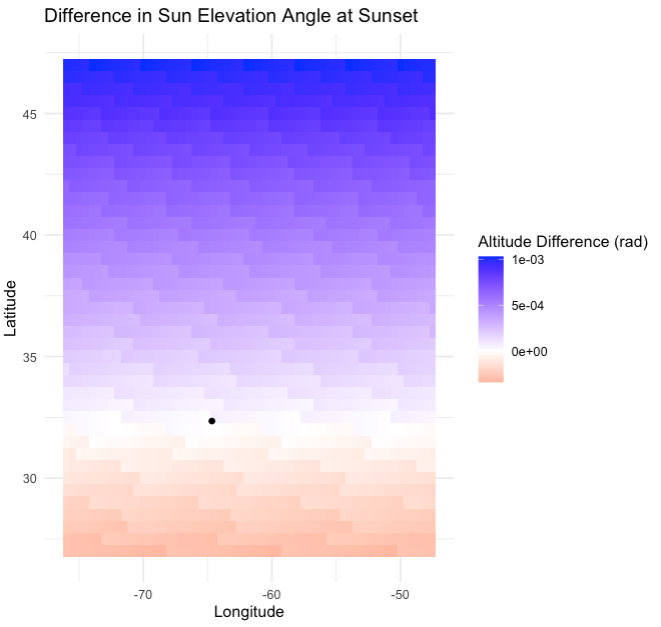


**Figure S3.** Differences in sun angle at sunset between Bermuda Island (32.347, -64.663 degrees used in the paper, black dot) and 2,378 locations within a 0.5-degree resolution grid. This grid covers the maximum latitudes and longitudes of the 90% population-level utilization distribution (UD) of GPS-tracked individuals (Fig. 5 in the main text). We calculated the differences for two key dates: at the start (January 23, 2023) and end (February 19, 2023) of the tracking period. Here we report the second date that showed the maximum differences between Bermuda and any point of the grid. We then extracted the maximum difference values (Max diff in sun angle at sunset = 0.001 rad = 0.058 degrees).


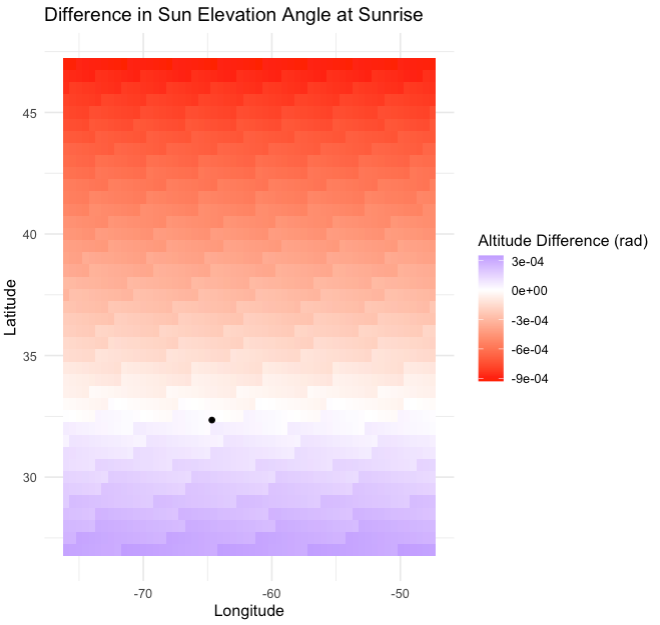


**Figure S4.** Differences in sun angle at sunrise between Bermuda Island (32.347, -64.663 degrees used in the paper, black dot) and 2,378 locations within a 0.5-degree resolution grid. This grid covers the maximum latitudes and longitudes of the 90% population-level utilization distribution (UD) of GPS-tracked individuals (Fig. 5 in the main text). We calculated the differences for two key dates: at the start (January 23, 2023) and end (February 19, 2023) of the tracking period. Here we report the second date that showed the maximum differences between Bermuda and any point of the grid. We then extracted the maximum difference values (Max diff in sun angle at sunrise = 0.0009 rad = 0.053 degrees).
